# Immune-Modulating Drug MP1032 with SARS-CoV-2 Antiviral Activity In Vitro: A potential Multi-Target Approach for Prevention and Early Intervention Treatment of COVID-19

**DOI:** 10.3390/ijms21228803

**Published:** 2020-11-20

**Authors:** Sara Schumann, Astrid Kaiser, Ferdinando Nicoletti, Katia Mangano, Paolo Fagone, Eduard van Wijk, Yu Yan, Petra Schulz, Beate Ludescher, Michael Niedermaier, Joerg von Wegerer, Pia Rauch, Christian Setz, Ulrich Schubert, Wolfgang Brysch

**Affiliations:** 1 MetrioPharm Deutschland GmbH, Am Borsigturm 100, 13507 Berlin, Germany; a.kaiser@metriopharm.com (A.K.); p.schulz@metriopharm.com (P.S.); b.ludescher@metriopharm.com (B.L.); m.niedermaier@metriopharm.com (M.N.); j.vonwegerer@metriopharm.com (J.v.W.); 2 Department of Biomedical and Biotechnological Sciences, University of Catania, 95123 Catania, Italy; ferdinic@unict.it (F.N.); kmangano@unict.it (K.M.); paolofagone@yahoo.it (P.F.); 3 Meluna Research, Department of Biophotonics, Koppelsedijk 1A, 4191 LC Geldermalsen, The Netherlands; evanwijk@melunaresearch.nl (E.v.W.); yan@melunaresearch.nl (Y.Y.); 4 Institute of Virology, Friedrich-Alexander University Erlangen-Nürnberg (FAU), 91054 Erlangen, Germany; pia.rauch@uk-erlangen.de (P.R.); christian.setz@uk-erlangen.de (C.S.); ulrich.schubert@fau.de (U.S.); 5 MetrioPharm AG, Bleicherweg, 10 8002 Zurich, Switzerland; w.brysch@metriopharm.com

**Keywords:** COVID-19, SARS-CoV-2, oxidative stress, inflammation, cytokine storm, drug development, COVID-19 drugs

## Abstract

At least since March 2020, the severe acute respiratory syndrome coronavirus type 2 (SARS-CoV-2) pandemic and the multi-organ coronavirus disease 2019 (COVID-19) are keeping a firm grip on the world. Although most cases are mild, older patients and those with co-morbidities are at increased risk of developing a cytokine storm, characterized by a systemic inflammatory response leading to acute respiratory distress syndrome and organ failure. The present paper focuses on the small molecule MP1032, describes its mode of action, and gives rationale why it is a promising option for the prevention/treatment of the SARS-CoV-2-induced cytokine storm. MP1032 is a phase-pure anhydrous polymorph of 5-amino-2,3-dihydro-1,4-phthalazinedione sodium salt that exhibits good stability and bioavailability. The physiological action of MP1032 is based on a multi-target mechanism including localized, self-limiting reactive oxygen species (ROS) scavenging activities that were demonstrated in a model of lipopolysaccharide (LPS)-induced joint inflammation. Furthermore, its immune-regulatory and PARP-1-modulating properties, coupled with antiviral effects against SARS-CoV-2, have been demonstrated in various cell models. Preclinical efficacy was elucidated in LPS-induced endotoxemia, a model with heightened innate immune responses that shares many similarities to COVID-19. So far, during oral clinical development with three-month daily administrations, no serious adverse drug reactions occurred, highlighting the outstanding safety profile of MP1032.

## 1. Introduction

The multi-organ coronavirus disease 2019 (COVID-19) is caused by infection with severe acute respiratory syndrome coronavirus type 2 (SARS-CoV-2). In susceptible patients, it may result in acute respiratory distress syndrome (ARDS), multiple system organ failure, and death. The key mechanism for this has been ascribed to a cytokine storm (also called cytokine release syndrome or macrophage overactivation syndrome), a systemic inflammatory response associated with an overstimulated immune system [1,2]. However, not all people exposed to SARS-CoV-2 are infected, and not all infected patients develop severe COVID-19. Among 1099 patients initially analyzed in Wuhan (China), 16% progressed to the severe phase showing respiratory failure requiring mechanical ventilation [3].

One of the most important unanswered question is: why do some people develop severe disease, whilst others do not? More and more data support the notion that there is a skewed mortality towards elderly men with co-morbidities [3,4]. Elderly persons often show a diminished/impaired pulmonary immunity with inadequate innate and adaptive immune responses. The so-called inflammaging is characterized by elevated levels of reactive oxygen species (ROS), inflammatory cytokines, and acute phase reactants (e.g., C-reactive protein) that lead to continuous low-grade inflammation accompanied by an overall decline in innate immune responses [5,6]. The process of inflammaging together with the presence of other age-related diseases, such as diabetes and cardiovascular diseases, is thought to be associated with increased severity and mortality of viral infections in elderly people [7].

SARS-CoV-2 infects the epithelial cells of the upper respiratory tract via the receptor for angiotensin-converting enzyme 2 (ACE2), triggering innate response mechanisms [8]; at later stages, the virus may disseminate to the lower respiratory tract [9]. Upon the entry of viral proteins and RNA into host cells, endoplasmic reticulum stress and mitochondrial dysfunction are induced, promoting ROS production [10]. Extensive ROS lead to oxidative stress and further increase cytokine production (e.g., IL-6), facilitating the dreaded cytokine storm, which drives severe COVID-19 cases. This is also a major factor for the witnessed multi-organ failure, ARDS, disseminated intravascular coagulation, and high mortality [11]. On a systemic level, high neutrophil-to-lymphocyte ratios are reported in critically ill COVID-19 patients and have been shown to predict in-hospital mortality [12]. It is hypothesized that this neutrophilia also generates excessive ROS (oxidative burst) that exacerbate the host immunopathological response resulting in a more severe disease [13]. 

At the cellular level, the maintenance of normal homeostasis and counteraction of detrimental ROS effects relies, in part, on effective DNA repair. Several DNA repair mechanisms exist, one of which is mediated by poly (ADP-ribose) polymerase 1 (PARP-1) that is responsible for ADP-ribosylation. PARP-1 is activated by binding to irregular/damaged DNA [14], which is often the result of extensive ROS production under these inflammatory conditions [15]. Hence, PARP enzymes are involved in a wide variety of cellular processes such as cell death, immune function, antiviral response, and autophagy [16]. In regard to SARS-CoV-2 infection, it is interesting that PARP-1 inhibition has been shown to limit inflammation-induced tissue damage, including acute lung injury in animal models [17], and PARP-1 inhibitors are therefore discussed as a potential treatment option for COVID-19 [16]. 

Similar to SARS-CoV and Middle East respiratory syndrome coronavirus (MERS-CoV), there is currently no clinically proven specific antiviral agent available for SARS-CoV-2 infection, and the medical need for safe, cost-effective, and easily distributable drugs is high. Our data suggest that the small molecule MP1032 exerts antiviral activity against SARS-CoV-2. MP1032 is a phase-pure anhydrous polymorph of 5-amino-2,3-dihydro-1,4-phthalazinedione sodium salt [18] that is very stable, water-soluble, and has good bioavailability via different routes of administration. It is currently under development at MetrioPharm (Zurich, Switzerland), reaching clinical Phase II for the oral treatment of psoriasis and showing an outstanding safety profile. The physiological action of MP1032 is based on a multi-target mechanism that depends on localized, self-limiting ROS scavenging activities and PARP-1-modulating properties. Efficacy has been demonstrated in preclinical models with excessively activated immune responses (such as lipopolysaccharide (LPS)-induced endotoxemia), suggesting MP1032 as a potential agent for the treatment/prevention of the SARS-CoV-2-induced cytokine storm. The present paper discusses this novel therapeutic agent, describes its mode of action, and offers a rationale for why it could be a promising treatment option for COVID-19.

## 2. Results

### 2.1. MP1032, a Self-Regulating, Highly Specific ROS Scavenger with PARP-1 Inhibitor Activity

Increased oxidative stress with excessive ROS production is a fundamental mechanism in inflammatory diseases (such as rheumatoid arthritis) and has also been proposed as a key COVID-19 pathway [19]. Based on its chemical properties, MP1032 is thought to scavenge excessive inflammation-induced ROS. During this reaction, MP1032 itself is oxidized, generating photon emission that is detectable by hypersensitive photon-counting devices, i.e., by the ultra-weak photon emission (UPE) technique [20]. 

**Figure 1:**
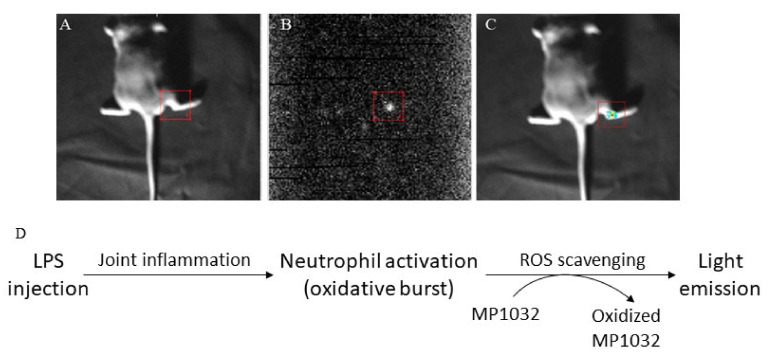
Effects of MP1032 on lipopolysaccharide (LPS)-induced production of reactive oxygen species (ROS). Using male DBA/1J mice, joint inflammation was induced in the right ankle by intra-articular injection of 10 µg LPS; as a control, PBS was injected into the left ankle joint. After two days, 100 mg/kg MP1032 was applied systemically via i.p. injection, and ultra-weak photon emission (UPE) was measured 15 min post-dose. (**A**) Position image, (**B**) photon image, (**C**) merged image with pseudo-color, and (**D**) proposed mechanism of ROS scavenging that leads to light emission detected by the UPE technique. Pictures show one representative mouse from a total of three mice.

To evaluate this ROS scavenging effect in detail, we used an arthritic LPS-induced inflammation model. Joint inflammation was induced in the right ankle of mice by intra-articular LPS injection. As a control, phosphate-buffered saline (PBS) was injected into the left ankle joint. At maximum inflammation, MP1032 was administered systemically via i.p. injection, and UPE was monitored. The scavenging reaction was observed between 1 min and 30 min after MP1032 injection, although it was strongest after 15 min. Figure 1 indicates MP1032-mediated photon emission in the right ankle joint, directly at the site of LPS-induced inflammation. In contrast, PBS application into the left ankle joint did not result in inflammation, and therefore no UPE was detectable. This unique and self-limiting mechanism is based on the fact that excessive ROS production creates intracellularly a highly localized alkaline environment [21], which deprotonates and thereby activates circulating MP1032 directly at the inflammatory site. After restoring the cellular physiologic redox balance via the scavenging of excessive ROS, the environment returns to normal pH, MP1032 action ceases, and the UPE signal declines. This signal decline is primarily due to the rapidly decreasing MP1032 activation, rather than to tissue drug concentrations, which persist considerably longer, as shown by extensive pharmacokinetic investigations made during regulatory research.

**Figure 2:**
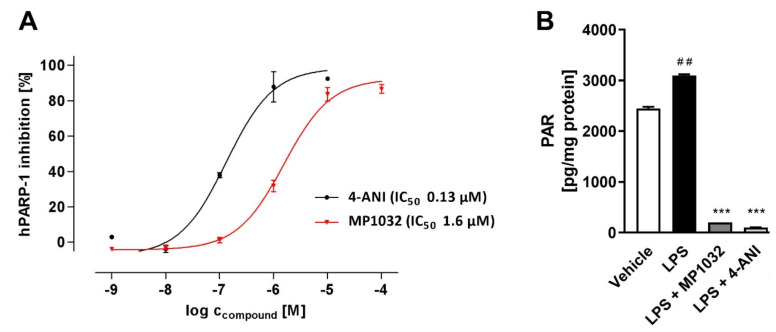
Effects of MP1032 on poly (ADP-ribose) polymerase 1(PARP-1). (**A**) Cell-free human PARP-1 enzyme assay with MP1032 or 4-amino-1,8-naphthalimide (4-ANI). Relative fluorescence units (RFU) raw data were normalized by using a NAD^+^ standard curve and expressed as inhibition of human PARP-1 (%). (**B**) Poly-ADP-ribosylation (PAR) of proteins in differentiated HL-60 cells upon treatment with 1 mM MP1032 or 50 μM 4-ANI. HL-60 were differentiated for 24 h with phorbol 12-myristate 13-acetate and then treated for another 24 h with MP1032 or 4-ANI under LPS inflammatory conditions. Total cellular proteins were isolated and subjected to PAR ELISA. PAR values were normalized to the respective protein content. Data are expressed as mean ± SEM, *n* = 4/group. Statistical analyses were performed using unpaired *T*-Test; ## *p* < 0.01 (Vehicle vs. LPS), *** *p* < 0.001 (treatments vs. LPS).

Oxidative stress induces DNA damage and thereby activates repair mechanisms such as PARP-1. Hence, further studies investigated whether MP1032 affects PARP-1 activity. Initially, a coupled enzyme assay with fluorometric read-out was used to determine the effect on human PARP-1 activity in a cell-free system. In principle, PARP activity leads to NAD^+^ depletion and reduced fluorescence, while reduced PARP-1 activity due to PARP inhibition is characterized by higher fluorescence values. For MP1032, a direct inhibitory activity on human PARP-1 was detected in the low micromolar range (IC_50_ = 1.55 μM), while the established PARP inhibitor 4-amino-1,8-naphthalimide (4-ANI) showed an IC_50_ of 0.13 μM (Figure 2A). In a second experiment, the PARP-1 inhibitory effect of MP1032 on differentiated HL-60 cells was explored. The amount of poly-ADP-ribosylated proteins was measured, and it was observed that differentiated but untreated HL-60 cells initially had high levels of PARP-1 activity (Figure 2B). Further, the data indicated that poly-ADP-ribosylation is not massively increased by LPS stimulation, an effect that was previously described [22]. Nevertheless, treating cells with 1 mM MP1032 or 50 μM 4-ANI dramatically decreased the amount of poly-ADP-ribosylation proteins by over 90%.

Overall, these studies demonstrate that MP1032 works as a ROS scavenger acting directly and exclusively at the site of inflammation. Furthermore, MP1032 seems to modulate PARP-1 activity directly and independently of its ROS scavenging capacities.

### 2.2. MP1032 Attenuates LPS-Induced Pro-Inflammatory Cytokine Activation In Vitro and In Vivo

During bacterial as well SARS-CoV-2-induced inflammation, oxidative stress and PARP-1 activation are tightly linked to the induction of pro-inflammatory cytokine production. This downstream activation of pro-inflammatory cytokines was investigated using LPS-stimulated HL-60 and murine/human primary immune cells incubated with MP1032.

**Figure 3:**
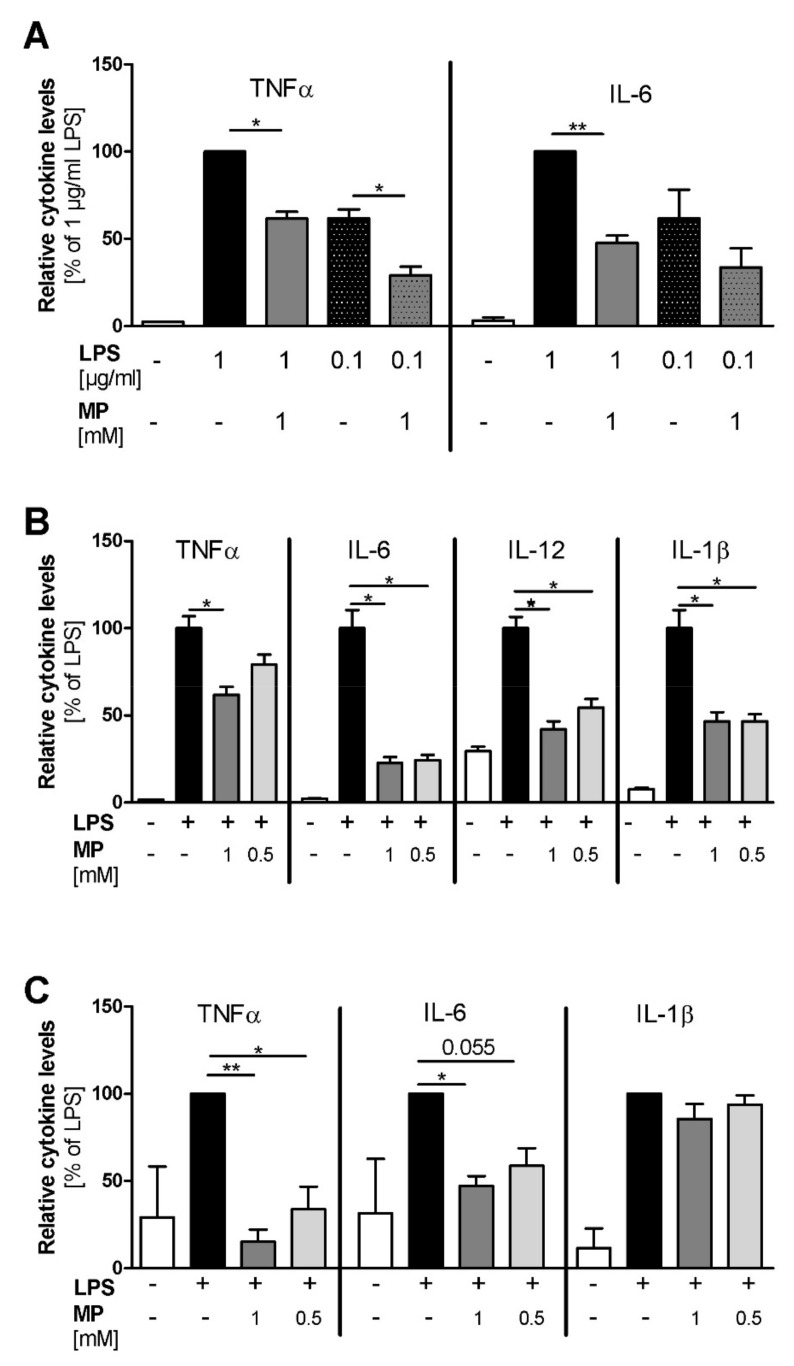
MP1032 attenuates LPS-induced cytokine release in vitro. (**A**) Human HL-60 cells were differentiated for 24 h using 50 ng/mL phorbol 12-myristate 13-acetate (PMA). One hour prior to LPS stimulation (with 0.1 µg/mL or 1 µg/mL LPS), differentiated cells were treated with 1 mM MP1032. (**B**) Murine peritoneal macrophages were isolated from female C57Bl/6 mice 4 days after i.p. injection of 3% thioglycolate medium. One hour prior to LPS stimulation (0.1 µg/mL), the isolated cells were pre-treated with 0.5 or 1 mM MP1032. (**C**) Human peripheral blood mononuclear cells were isolated from blood buffy coats by density gradient centrifugation. One hour prior to stimulation with 0.1 µg/mL LPS, the cells were treated with 0.5 or 1 mM MP1032. Cell-free supernatants were collected from all cell types after 24 h, and secreted cytokine levels were detected by ELISA. Data are expressed as mean ± SEM relative to the LPS-treated vehicle group that was normalized to 100%. (**A**) *n* = 2, (**B**) *n* = 2 (three mice pooled in each experiment), (**C**) *n* = 3. Statistical analyses were performed using unpaired *T*-Test with (**C**) or without (**A**/**B**) Welch’s correction; * *p* < 0.05, ** *p* < 0.01.

The human promyelocytic leukemia cell line HL-60 was differentiated along the macrophage/monocyte lineage, and LPS stimulation with either 0.1 µg/mL or 1 µg/mL LPS led to dose-dependent TNFα and IL-6 induction (Figure 3A). Pre-treatment with 1 mM MP1032 reduced the secretion of both cytokines. Using 1 µg/mL LPS, MP1032 reduced the TNFα and IL-6 levels about 40% and 50%, respectively. When 0.1 µg/mL LPS was used, MP1032 led mainly to a decrease of TNFα (about 50%).

Using LPS-stimulated murine peritoneal macrophages (PM), we assessed the effects of MP1032 in a primary cell model. While MP1032 did not affect cytokine secretion in unstimulated murine PMs, LPS-induced pro-inflammatory cytokine concentrations were reduced by MP1032 pre-treatment (Figure 3B). In detail, 1 mM MP1032 significantly reduced the levels of TNFα, IL-6, IL12, and IL-1β by about 40%, 80%, 60%, and 50%, respectively. Interestingly, for all cytokines, similar MP1032 effects were observed after 48 h and 72 h of culture. Furthermore, these effects were validated in PMs of a second mouse strain (Balb/c), and a clear dose-dependency was observed with six different MP1032 concentrations ranging from 1 mM to 0.25 mM (Supplementary Figure S1). 

Using human peripheral blood mononuclear cells (PBMCs), MP1032 at concentrations of 0.5 mM and 1 mM reduced LPS-induced TNFα and IL-6 secretion in a dose-dependent manner (Figure 3C). IL-1β levels were reduced to a much lower extent, not reaching statistical significance. Notably, the oxidized form of MP1032 (3-aminophthalic acid sodium salt) did not have any effect on LPS-induced TNFα and IL-6 secretion, indicating that the ROS scavenging properties previously described and an intact structure are mandatory to achieve suppression of pro-inflammatory cytokine production.

**Figure 4:**
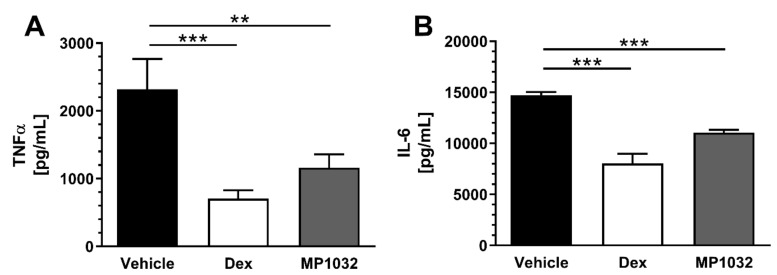
Pro-inflammatory plasma cytokine levels in LPS-treated mice. Fifteen minutes prior to LPS challenge (300 µg/mouse; i.p.), female CD1 mice were treated via i.p. injection with 0.5 mg/kg MP1032. Dexamethasone (Dex; 0.3 mg/kg) was dosed twice, 24 h and 1 h before LPS application; as a vehicle, water for injection was used. Mice were sacrificed 2 h after the LPS challenge, and plasma TNFα (**A**) and IL-6 (**B**) concentrations were determined by ELISA. Data are expressed as mean ± SEM, *n* = 9–10/group. Statistical analyses were performed using the Mann–Whitney test; ** *p* < 0.01, *** *p* < 0.001.

MP1032 efficacy in inhibiting the secretion of pro-inflammatory cytokines in vitro prompted us to evaluate its in vivo efficacy. The used sublethal LPS-challenged mouse model represents a model of endotoxic shock that is pathophysiologically characterized by dysregulated secretion of pro-inflammatory cytokines [23]. As depicted in Figure 4, MP1032 pre-treatment significantly reduced plasma cytokine levels compared to vehicle-treated mice by 50% (TNFα) and 25% (IL-6). The positive control, dexamethasone, was administered two times (instead of once as for MP1032) and reduced TNFα as well as IL-6 concentrations by 70% and 45%, respectively.

Taken together, MP1032 reduces pro-inflammatory cytokine levels in various immune cells and attenuates LPS endotoxemia-induced cytokine release in vivo.

### 2.3. MP1032 Exhibits Antiviral Activity against SARS-CoV-2

**Figure 5:**
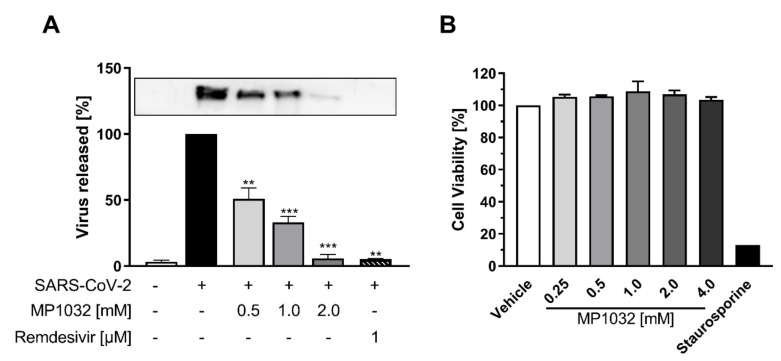
Influence of MP1032 on SARS-CoV-2 replication and viability of Vero B4 cells. Confluent monolayers of Vero B4 cells were infected with a 100-fold dilution of the field isolate SARS-CoV-2_PR-1_ for 1 h and afterwards treated with MP1032. (**A**) SARS-CoV-2_PR-1_ nucleocapsid was determined in supernatants by Western Blot analysis 72 h post-infection; remdesivir (1 µM) was used as a positive control (a representative blot is shown). (**B**) The viability of uninfected and treated cells was assessed by the water-soluble tetrazolium salt 1 assay; staurosporine (1 µM) served as a positive control. Data are expressed as mean ± SEM; (**A**) *n* = 4/group (remdesivir *n* = 2); (**B**) *n* = 3/group. Statistical analyses were performed using unpaired *T*-Test with Welch’s correction; ** *p* < 0.01, *** *p* < 0.001.

To analyze if MP1032 possesses antiviral activity against SARS-CoV-2, Vero B4 cells were infected with SARS-CoV-2_PR-1_ and treated with MP1032. Three days post infection, cell culture supernatants were harvested, and virus production was analyzed by Western blot. Treatment with MP1032 led to a significant reduction of SARS-CoV-2 replication. At 1 mM MP1032, the production of progeny virions was inhibited by about 70% and at 2 mM, it was almost completely inhibited (Figure 5A). To control for potential unspecific effects on cell viability, a water-soluble tetrazolium salt 1 (WST-1) assay was performed in uninfected Vero B4 cells, showing that MP1032 concentrations effectively suppressing SARS-CoV-2 replication did not affect cell viability (Figure 5B). 

These cumulative data demonstrate that MP1032 exhibits strong antiviral activity against SARS-CoV-2, while having no impact on Vero B4 cell viability.

### 2.4. Clinical Safety Data

**Figure 6:**
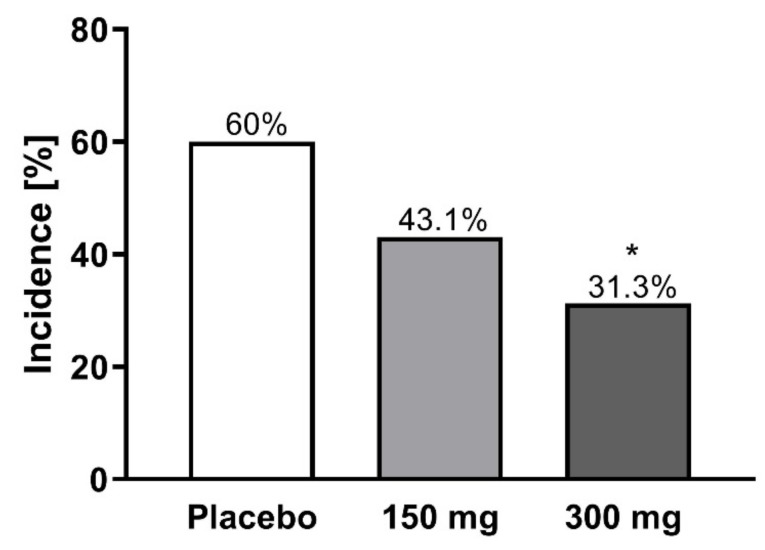
Incidence of treatment-emergent adverse events (TEAEs) in a clinical phase II study. In total, 155 patients with moderate to severe chronic plaque psoriasis were randomized, receiving 300 mg MP1032, 150 mg MP1032, or placebo twice daily for a period of 12 weeks (EudratCT-No:2017-003484-36). Adverse events were coded according to the Medical Dictionary for Regulatory Activities. Statistical analyses were performed using two-sided Fisher’s exact test; * *p* < 0.05.

The data presented above clearly indicate that MP1032 is a promising treatment option for SARS-CoV-2-infection and the attenuation of COVID-19 inflammatory pathophysiology. To assess the feasibility of using this drug, especially in at-risk COVID-19 patients, clinical safety data obtained from a randomized, double-blind, placebo-controlled phase II study in psoriasis patients are presented here. In total, 155 patients were randomized, receiving 300 mg MP1032, 150 mg MP1032, or placebo twice daily over a study period of 12 weeks. Safety evaluation revealed that MP1032 at both doses was safe and generally well-tolerated, with no clinically important safety issues being identified. No deaths occurred, and only three treatment-emergent serious adverse events (SAEs) were reported (all in the placebo group). Furthermore, most of the treatment-emergent adverse events (TEAEs) were “unlikely” or “not related” to MP1032, and patients receiving 300 mg MP1032 showed a statistically significant reduction in the incidence of TEAEs compared to patients receiving placebo (Figure 6). 

To summarize, MP1032 was successfully evaluated in the clinical setting, showing an exceptional safety profile with good tolerability.

## 3. Discussion

The World Health Organization declared the COVID-19 outbreak, caused by SARS-CoV-2, a pandemic on 11 March 2020. Given this immense health risk, several drugs have been clinically tested, ranging from antivirals, antibiotics, biologics, and corticosteroids up to antioxidants [24,25]. Despite this, and a massive international initiative to develop SARS-CoV-2 vaccines, there still remains an unabated, urgent need for effective pharmacologic interventions that prevent COVID-19 patients from clinical declining towards the need for intensive care and assisted ventilation. As of this writing, no such safe, convenient, and widely applicable treatment meeting these criteria has been identified or approved.

Here, we present data on MP1032, a small-molecule drug that combines localized, auto-regulated ROS scavenging and immune-modulating effects with specific antiviral properties against SARS-CoV-2. 

To date, 146 subjects received MP1032 during oral clinical development up to phase II (EudraCT-Nos. 2014-004606-15, 2015-005159-28, 2017-003484-36). Besides a demonstrated trend of a dose-dependent anti-inflammatory effect in the exploratory psoriasis trials, no serious adverse events occurred in the MP1032 treatment groups, highlighting its outstanding safety profile (all reported adverse drug reactions are summarized in Supplementary Table S1).

The pronounced ROS scavenging and immune-modulating properties of MP1032 originally prompted its development in the field of autoimmune inflammatory diseases (like psoriasis and rheumatoid arthritis) as well as age-related degenerative diseases caused by chronic low-grade inflammation (inflammaging). Alveolar inflammation and acute lung injury in COVID-19 are driven by similar cellular pathologies, particularly with respect to oxidative stress and the pattern of cytokines involved in the cytokine release syndrome [26,27].

Several antibodies directed against these pro-inflammatory cytokines or their respective receptors have already been evaluated in the clinical setting, so far with moderate success only. This may be due to the fact that these biologics suppress the host immune system, which is detrimental in the early non-severe stages where the adaptive immune response helps to eliminate the virus and protects against disease progression to severe stages [28]. The importance of treatment timing with immune suppressants was demonstrated by the RECOVERY trial. In this study, dexamethasone resulted in lower mortality among severe COVID-19 cases who were receiving respiratory support, while in patients who were not receiving invasive mechanical ventilation, dexamethasone treatment was harmful and resulted in a higher mortality rate [29]. In contrast to immune-suppressing biologics or corticosteroids, MP1032 acts as an immune-modulating drug, since it does not completely suppress cytokine induction, which serves an important role during the anti-infective response. The redox-balancing mechanism of MP1032 rather curtails the overshoot of the immune response. Such a pharmacodynamic profile lends itself for an early intervention treatment, where preserving a physiologic immune response and inhibiting an overshoot are essential for preventing disease progression. 

Similar to chronic inflammatory diseases, several respiratory viruses induce a dysregulated ROS formation by disrupting antioxidant mechanisms leading to an unbalanced oxidant/antioxidant ratio and subsequent oxidative cell damage [30]. The early use of antioxidants, such as vitamin C and *N*-acetyl cysteine, has therefore been discussed as a potential option to prevent/treat COVID-19 [31,32], and several studies are currently ongoing (e.g., ClinicalTrials.gov Identifier: NCT04344184, NCT04323514, NCT04374461, NCT04419025). In contrast to these antioxidants, which at high concentrations tend to scavenge even physiologically necessary ROS, MP1032 action is self-regulating and highly localized. This unique mechanism is based on the fact that excessive ROS production creates intracellularly a highly localized alkaline environment [21]. Due to its chemical properties, circulating MP1032 is activated by this pH shift only at the inflammatory site. As MP1032 scavenges excessive ROS, the pH normalizes, and MP1032 activation shuts down. Hence, excessive ROS are scavenged, but physiologically necessary ROS levels are spared, ensuring that there is no impairment of cellular signaling.

In vitro evaluation of antiviral activity directed against SARS-CoV-2 is preferably performed in Vero cells [33]. Using this model, we could show that MP1032 suppresses virus replication in a dose-dependent manner, while it has no effect on cell viability. The molecular basis for this antiviral effect still needs to be fully elucidated. It is conceivable that MP1032 inhibits virus entry via acting on the ACE2 receptor, as it was proposed for *N*-acetyl cysteine [34]. MP1032 could further attenuate prolonged virus replication by preventing oxidative stress [35] or by limiting ADP ribosylation of the viral nucleocapsid protein via PARP-1 inhibition [36]. Additional studies should be conducted to evaluate this in more detail.

## 4. Materials and Methods

### 4.1. MP1032

MP1032 (5-amino-2,3-dihydro-1,4-phthalazinedione sodium salt) polymorph was synthesized by a specialized manufacturer (ChemCon, Freiburg i. Br., Germany) in a multi-step procedure. The polymorph was formed in the last reaction step, and its polymorphic purity was confirmed by X-ray powder diffraction as previously described [18].

### 4.2. In Vitro Studies

#### 4.2.1. HL-60 Cells

As described by Xu et al. [37], the human leukemic cell line HL-60 was differentiated using 50 ng/mL phorbol 12-myristate 13-acetate (PMA, Sigma-Aldrich, Darmstadt, Germany) for 24 h. To confirm successful differentiation along the monocytic/macrophage lineage, HL-60 were stained according to the method of Pappenheim [38]. One hour prior to LPS stimulation (with 0.1 µg/mL or 1 µg/mL LPS; Sigma-Aldrich, Darmstadt, Germany), differentiated cells were treated with 1 mM MP1032 (or 50 µM 4-ANI for PARP-1 activity assay). After 24 h, cell-free supernatants were collected and stored at −20 °C for cytokine quantification, while the cells were lysed for PARP-1 assay.

#### 4.2.2. Murine Peritoneal Macrophages

Female C57Bl/6 mice (6–8 weeks old) were purchased from Envigo (San Pietro al Natisone, Italy) and housed under standard laboratory conditions (specific-pathogen-free) at the animal facility of the University of Catania (Catania, Italy). This experiment was performed in accordance with the Directive 2010/63/UE (enforced by the Italian D. Lgs 26/2014); physical facilities and equipment for accommodation and care of animals were in accordance with the provisions of EEC Council Directive 86/609. Food and water were applied ad libitum. Animal handling and the study protocol were in accordance with international guidelines and approved by the local Institutional Animal Care and Use Committee and the Ministry of Health (project identification code: 388 of 8 May 2015).

Four days after i.p. injection of 3% thioglycolate medium (*w*/*v* in distilled water; St Louis, MO, USA), murine peritoneal macrophages were isolated according to a published protocol [39]. Briefly, cells were collected by injecting 10 mL of complete medium (DMEM/F12 Medium containing 10% fetal calf serum) into the peritoneal cavity using a 30 cc syringe attached to a 19-gauge needle, followed by slowly withdrawing the lavage fluid. The cells were washed twice in PBS and counted. A total of 3–4 × 10^6^ macrophages were collected from each mouse.

Cell purity was confirmed by flow cytometry (using CD14 expression), and isolated PMs were allowed to adhere overnight. One hour prior to LPS stimulation (0.1 µg/mL; *Escherichia coli* O55:B5, St Louis, MO, USA), the cells (0.5 × 10^6^ cells/well in 24-well plates with 1 mL total volume of medium) were pre-treated with scalar MP1032 concentrations (ranging from 1 mM to 0.025 mM). Two independent experiments (each using cells pooled from three mice) were performed, and secreted cytokine levels were determined 24, 48, and 72 h after LPS stimulation using the ELISA technique.

#### 4.2.3. Human Peripheral Blood Mononuclear Cells

PBMCs were isolated from buffy coats of three different healthy blood donors (Institute for transfusion medicine, Suhl, Germany) by density gradient centrifugation (2000 rpm, room temperature, 15 min, using Ficoll Hypaque, density 1.077 g/mL; Biochrome, Berlin, Germany). The PBMC fraction was removed, repeatedly washed with PBS, and finally resolved in Roswell Park Memorial Institute (RPMI) medium containing 10% (*v*/*v*) fetal calf serum (FCS), 2 mM L-glutamine, 100 U/mL penicillin, and 100 μg/mL streptomycin. One hour prior to stimulation with 0.1 µg/mL LPS (from *Escherichia coli* O111:B4; Sigma Aldrich, Taufkirchen, Germany), PBMCs were treated with different concentrations of MP1032 (1 mM and 0.5 mM). After LPS stimulation, the PBMCs were incubated for 24 h. Finally, the supernatants of the PBMC culture were collected and frozen at −20 °C until cytokine concentrations were determined by ELISA.

#### 4.2.4. Vero B4 Cell Culture and Infection

Vero B4 cells (National institute of health, USA) were maintained in Dulbecco’s Modified Eagle’s Medium (DMEM) containing 10% (*v*/*v*) inactivated FCS, 2 mM L-glutamine, 100 U/mL penicillin, and 100 μg/mL streptomycin. Confluent monolayers of Vero B4 cells were infected in FCS-free DMEM with a 100-fold dilution of the field isolate SARS-CoV-2_PR-1_ (isolated from a 61-year-old patient 6 days after the presumed date of infection and 2 days after the start of mild COVID 19 symptoms; see [40]) for 1 h and afterwards treated with MP1032 or remdesivir (1 µM; Hoelzel Biotech, Köln, Germany). Virus-containing cell culture supernatants were harvested 72 h post-infection, and released virions were purified through a 20% (*w*/*v*) sucrose cushion (20,000× *g*, 4 °C, 90 min). After washing with PBS, the pellet was dissolved in SDS sample buffer and used for Western Blot analysis. The viability of uninfected and treated cells was assessed by the WST-1 assay (Roche, Switzerland) according to the manufacturer’s instructions using staurosporine (1 µM; Sigma-Aldrich, Darmstadt, Germany) as a positive control.

### 4.3. In Vivo Studies

#### 4.3.1. Intra-Articular Inflammation and Ultra-Weak Photon Emission Imaging

The study was performed at the animal facility of the Changchun University of Chinese Medicine. The animals were kept under standard laboratory conditions (water and food ad libitum). The animals were single-housed according to the Chinese legislation; animal handling and the study protocol were in accordance with international guidelines and approved by the local Institutional Animal Care and Use Committee. Cages were sterilized and filled with wood shavings and cellulose as bedding material. Automatically controlled environmental conditions were set to maintain the temperature at 20–24 °C with a relative humidity of 50–60%; 12 h light–dark cycle.

Male DBA/1J mice aged 6–7 weeks received intra-articular injection of LPS (1 × 10^−5^ g LPS solubilized in 20 μL PBS) into the right ankle joint through the Achilles tendon using a 30-gauge needle, while an identical volume of PBS was injected into the left ankle joint. After two days, the mice received i.p. injection of 2.5 mg MP1032, which corresponds to a final dose of 100 mg/kg b.w. UPE imaging using a highly sensitive electron-multiplying CCD camera system (iXon 888 EM+; Andor Technology Ltd, Belfast, Northern Ireland) was performed 15 min after MP1032 injection: the mice were anesthetized using a Matrx VMR small animal anesthesia device (Midmark, Miamisburg, OH, USA) via isoflurane vapor (3% during induction and 2% during the experiment); the duration of anesthesia was kept as short as possible (max. 35 min). A position image of each mouse was taken, and after removing the red-light torch, UPE images were recorded in complete darkness.

#### 4.3.2. Sublethal LPS Model

This experiment was performed in accordance with the Directive 2010/63/UE (enforced by the Italian D. Lgs 26/2014) at the animal facility of the University of Catania (Catania, Italy); physical facilities and equipment for accommodation and care of animals were in accordance with the provisions of EEC Council Directive 86/609. The animals were kept under standard laboratory conditions (non-specific-pathogen-free) in a climate-controlled room (20–24 °C, relative air humidity 30–70%) with a 12 h light–dark cycle; food and water were applied ad libitum. CD1 mice (female, 6 weeks old) were purchased from Harlan Laboratories (San Pietro al Natisone, Italy). They were allowed to adapt to the animal facility for at least one week before commencing the study. Fifteen minutes prior to i.p. LPS challenge (300 µg/mouse; from *Salmonella enterica*, Cod L6011, Sigma, Milano, Italy), the mice were treated via i.p. injection with 0.5 mg/kg MP1032. As a control, dexamethasone (Dex; 0.3 mg/kg, Soldesam, Labotatorio Biologico Milanese, Varese, Italy) was dosed twice, 24 h and 1 h before LPS application; as a vehicle, water for injection was used. Mice were sacrificed by CO_2_ inhalation 2 h after the LPS challenge, blood was collected the via cava vein, and plasma was obtained by centrifugation at 2000 rpm for 10 min. Plasma TNFα and IL-6 concentrations were determined by ELISA.

### 4.4. Clinical Phase II Trial

In a randomized, double-blind, placebo-controlled study (EudratCT-No:2017-003484-36), two oral doses of MP1032 were evaluated over a period of 12 weeks in patients with moderate to severe chronic plaque psoriasis (PASI 10–20). In total, 155 patients were randomized, receiving 300 mg MP1032 (48 patients), 150 mg MP1032 (52 patients), or placebo (55 patients) twice daily. For safety evaluation, adverse events were recorded systemically according to the protocol and coded according to the Medical Dictionary for Regulatory Activities (MedDRA).

### 4.5. Cytokine Measurement

To measure cytokine concentration in cell culture supernatants, ELISA kits from BioLegend (San Diego, CA, USA) were used for the human HL-60 cells/PMBCs and from LifeSpan BioSciences, Inc. (Seattle, WA, USA) for the murine PMs. Plasma TNFα and IL-6 levels from the sublethal LPS model were determined using eBioscience or Bender Medsystem kits (Prodotti Gianni, Milano, Italy). ELISA assays were performed according to the manufacturer’s instructions. Data of the in vitro studies are expressed relative to the LPS-treated vehicle group that was normalized to 100%.

### 4.6. PARP-1 Activity in HL-60 Cells

PARP-1 activity in human HL-60 cells was determined using the PARP in vivo Pharmacodynamic Assay II (Trevigen, Gaithersburg, MD, USA) according to the manufacturer’s instructions. Poly-ADP-ribosylation was normalized to the protein content measured in the cell lysates.

### 4.7. Cell-Free PARP-1 Enzyme Assay

Cell-free human PARP-1 enzyme activity was determined in a coupled enzyme assay with fluorometric read-out. In a first step, 200 nM NAD^+^ were mixed with the PARP mix consisting of 1.25 μg activated (partly cleaved) DNA (Sigma#D4522) and 1.0 U/well hPARP-1 (Trevigen #4668-100-01) in assay buffer (50 mM Tris and 2 mM MgCl_2_ (pH 8.0)). Subsequently, MP1032 or 4-ANI prepared in assay buffer were added to the reaction mixture. Each assay was performed with a NAD^+^ standard curve (0–100 nM NAD^+^) and a PARP minus control for data analysis. After 90 min, an alcohol dehydrogenase/diaphorase mix consisting of 50 mU alcohol dehydrogenase, 5 mU diaphorase, 50 μM resazurin, and 2% EtOH (*v*/*v*) was added. Fluorescence kinetics (5 min steps for 40 min) was measured using a Synergy2 plate reader (BioTek, Bad Friedrichshall, Germany) with λ_exc_ 540/25 nm, λ_em_ 590/20 nm, and a 550 nm mirror. Data were calculated using NAD^+^ standard curve and expressed as percent hPARP-1 inhibition.

### 4.8. SDS-PAGE and Western Blotting

Protein samples were separated by SDS-PAGE, transferred onto nitrocellulose membranes, blocked with 3% bovine serum albumin, and incubated with the appropriate primary antibody. The SARS-CoV-2 nucleocapsid antibody (GTX135357; Genetex, Eching, Germany) and an anti-rabbit secondary antibody coupled to horseradish peroxidase (Dianova, Hamburg, Germany) were used.

### 4.9. Statistical Analyses

All data are presented as mean ± SEM. Statistical calculation was performed using GraphPad Prism (GraphPad Software, La Jolla, CA, USA). Normal distribution was tested using the Kolmogorov–Smirnov test. To assess the direct treatment effects vs. those of the vehicle group, unpaired *T*-Test with Welch’s correction was used to evaluate cytokine secretion from PBMCs and SARS-CoV-2 replication, while *T*-Test without Welch’s correction was used to analyze poly-ADP-ribosylation of proteins in HL-60 cells and cytokine secretion from HL-60 cells/PMs. Mann–Whitney Test was used to evaluate plasma cytokine levels from the sublethal LPS model. To assess the incidence of treatment-emergent adverse events in the clinical phase II study, two-sided Fisher’s exact test was used. Differences with *p* < 0.05 were considered statistically significant.

## 5. Conclusions

No orally available, clinically proven antiviral agent is currently accessible for SARS-CoV-2 infection, and thus the medical need for new, safe, effective, and easily distributable drugs remains high. Here, we show that MP1032, a phase-pure anhydrous polymorph of 5-amino-2,3-dihydro-1,4-phthalazinedione sodium salt exerts immune-modulatory, self-regulated ROS scavenging, and SARS-CoV-2 antiviral properties. This pharmacodynamic profile, which simultaneously addresses several crucial pathophysiological processes of SARS-CoV-2 infection and COVID-19 renders MP1032 a promising candidate for the treatment and possibly prevention of COVID-19. As a next step, this hypothesis should be tested in a controlled clinical trial.

## Supplementary Materials

Supplementary Materials can be found at https://www.mdpi.com/1422-0067/21/22/8803/s1.

## Abbreviations

4-ANI	4-amino-1,8-naphthalimide
ACE2	Angiotensin-converting enzyme 2
ARDS	Acute respiratory distress syndrome
COVID-19	Corona virus disease 2019
CD14	Cluster of differentiation 14
Dex	Dexamethasone
DMEM	Dulbecco’s Modified Eagle’s Medium
ELISA	Enzyme-linked Immunosorbent Assay
EtOH	Ethanol
FCS	Fetal calf serum
IL	Interleukin
LPS	Lipopolysaccharide
MedDRA	Medical Dictionary for Regulatory Activities
PARP1	Poly (ADP-ribose) polymerase 1
PASI	Psoriasis Area and Severity Index
PBMC	Peripheral blood mononuclear cells
PBS	Phosphate-buffered saline
PM	Peritoneal macrophages
PMA	Phorbol 12-myristate 13-acetate
RFU	Relative fluorescence units
ROS	Reactive oxygen species
RPMI	Roswell Park Memorial Institute medium
SAE	Serious adverse event
SARS-CoV-2	Severe acute respiratory syndrome coronavirus type 2
SDS-PAGE	Sodium dodecyl sulfate polyacrylamide gel electrophoresis
SEM	Standard error of the mean
TEAE	Treatment-emergent adverse event
TNFα	Tumor necrosis factor-alpha
UPE	Ultra-weak photon emission
WST-1	Water-soluble tetrazolium salt 1 assay

## References

[1] Huang C., Wang Y., Li X., Ren L., Zhao J., Hu Y., Zhang L., Fan G., Xu J., Gu X. (2020). Clinical features of patients infected with 2019 novel coronavirus in Wuhan, China. Lancet.

[2] Li X., Geng M., Peng Y., Meng L., Lu S. (2020). Molecular immune pathogenesis and diagnosis of COVID-19. J. Pharm. Anal..

[3] Guan W.-J., Ni Z.-Y., Hu Y., Liang W.-H., Ou C.-Q., He J.-X., Liu L., Shan H., Lei C.-L., Hui D.S.C. (2020). Clinical Characteristics of Coronavirus Disease 2019 in China. N. Engl. J. Med..

[4] Williamson E.J., Walker A.J., Bhaskaran K., Bacon S., Bates C., Morton C.E., Curtis H.J., Mehrkar A., Evans D., Inglesby P. (2020). Factors associated with COVID-19-related death using OpenSAFELY. Nature.

[5] Lowery E.M., Brubaker A.L., Kuhlmann E., Kovacs E.J. (2013). The aging lung. Clin. Interv. Aging.

[6] Nunn A., Guy G., Brysch W., Botchway S., Frasch W., Calabrese E., Bell J. (2020). SARS-CoV-2 and mitochondrial health: Implications of lifestyle and ageing. Immun. Ageing.

[7] Nasi A., McArdle S., Gaudernack G., Westman G., Melief C., Rockberg J., Arens R., Kouretas D., Sjölin J., Mangsbo S. (2020). Reactive oxygen species as an initiator of toxic innate immune responses in retort to SARS-CoV-2 in an ageing population, consider N-acetylcysteine as early therapeutic intervention. Toxicol. Rep..

[8] Li F., Li W., Farzan M., Harrison S.C. (2005). Structure of SARS coronavirus spike receptor-binding domain complexed with receptor. Science.

[9] Zhou F., Yu T., Du R., Fan G., Liu Y., Liu Z., Xiang J., Wang Y., Song B., Gu X. (2020). Clinical course and risk factors for mortality of adult inpatients with COVID-19 in Wuhan, China: A retrospective cohort study. Lancet.

[10] Shi C.-S., Nabar N.R., Huang N.-N., Kehrl J.H. (2019). SARS-Coronavirus Open Reading Frame-8b triggers intracellular stress pathways and activates NLRP3 inflammasomes. Cell Death Discov..

[11] Shi Y., Wang Y., Shao C., Huang J., Gan J., Huang X., Bucci E., Piacentini M., Ippolito G., Melino G. (2020). COVID-19 infection: The perspectives on immune responses. Cell Death Differ..

[12] Fu J., Kong J., Wang W., Wu M., Yao L., Wang Z., Jin J., Wu D., Yu X. (2020). The clinical implication of dynamic neutrophil to lymphocyte ratio and D-dimer in COVID-19: A retrospective study in Suzhou China. Thromb. Res..

[13] Laforge M., Elbim C., Frère C., Hémadi M., Massaad C., Nuss P., Benoliel J.-J., Becker C. (2020). Tissue damage from neutrophil-induced oxidative stress in COVID-19. Nat. Rev. Immunol..

[14] Bai P. (2015). Biology of Poly(ADP-Ribose) Polymerases: The Factotums of Cell Maintenance. Mol. Cell.

[15] Bai P., Virág L. (2012). Role of poly(ADP-ribose) polymerases in the regulation of inflammatory processes. FEBS Lett..

[16] Curtin N., Bányai K., Thaventhiran J., Le Quesne J., Helyes Z., Bai P. (2020). Repositioning PARP inhibitors for SARS-CoV-2 infection(COVID-19); a new multi-pronged therapy for acute respiratory distress syndrome?. Br. J. Pharmacol..

[17] Pazzaglia S., Pioli C. (2019). Multifaceted Role of PARP-1 in DNA Repair and Inflammation: Pathological and Therapeutic Implications in Cancer and Non-Cancer Diseases. Cells.

[18] Martin T., Greim D., Milius W., Niedermaier M., Ludescher B., von Wegerer J., Brysch W., Bärwinkel K., Senker J., Breu J. (2015). The Same at a First Glance: The Diffractogram of a New Polymorph of Anhydrous Sodium Luminolate Almost Perfectly Resembles the Diffraction Trace of an Already Known Polymorph. Z. Anorg. Allg. Chem..

[19] Delgado-Roche L., Mesta F. (2020). Oxidative Stress as Key Player in Severe Acute Respiratory Syndrome Coronavirus (SARS-CoV) Infection. Arch. Med. Res..

[20] Cifra M., Pospíšil P. (2014). Ultra-weak photon emission from biological samples: Definition, mechanisms, properties, detection and applications. J. Photochem. Photobiol. B Biol..

[21] Ikebuchi Y., Masumoto N., Tasaka K., Koike K., Kasahara K., Miyake A., Tanizawa O. (1991). Superoxide anion increases intracellular pH, intracellular free calcium, and arachidonate release in human amnion cells. J. Biol. Chem..

[22] Bhatia M., Kirkland J.B., Meckling-Gill K.A. (1995). Modulation of poly(ADP-ribose) polymerase during neutrophilic and monocytic differentiation of promyelocytic (NB4) and myelocytic (HL-60) leukaemia cells. Biochem. J..

[23] Nicoletti F., Mancuso G., Cusumano V., Di Marco R., Zaccone P., Bendtzen K., Teti G. (1997). Prevention of endotoxin-induced lethality in neonatal mice by interleukin-13. Eur. J. Immunol..

[24] Fajgenbaum D.C., Khor J.S., Gorzewski A., Tamakloe M.-A., Powers V., Kakkis J.J., Repasky M., Taylor A., Beschloss A., Hernandez-Miyares L. (2020). Treatments Administered to the First 9152 Reported Cases of COVID-19: A Systematic Review. Infect. Dis. Ther..

[25] Costela-Ruiz V.J., Illescas-Montes R., Puerta-Puerta J.M., Ruiz C., Melguizo-Rodríguez L. (2020). SARS-CoV-2 infection: The role of cytokines in COVID-19 disease. Cytokine Growth Factor Rev..

[26] Schett G., Manger B., Simon D., Caporali R. (2020). COVID-19 revisiting inflammatory pathways of arthritis. Nat. Rev. Rheumatol..

[27] Tan Y., Qi Q., Lu C., Niu X., Bai Y., Jiang C., Wang Y., Zhou Y., Lu A., Xiao C. (2017). Cytokine Imbalance as a Common Mechanism in Both Psoriasis and Rheumatoid Arthritis. Mediat. Inflamm..

[28] Cain D.W., Cidlowski J.A. (2020). After 62 years of regulating immunity, dexamethasone meets COVID-19. Nat. Rev. Immunol..

[29] (2020). Dexamethasone in Hospitalized Patients with Covid-19—Preliminary Report. N. Engl. J. Med..

[30] Gjyshi O., Bottero V., Veettil M.V., Dutta S., Singh V.V., Chikoti L., Chandran B. (2014). Kaposi’s sarcoma-associated herpesvirus induces Nrf2 during de novo infection of endothelial cells to create a microenvironment conducive to infection. PLoS Pathog..

[31] Cheng R.Z. (2020). Can early and high intravenous dose of vitamin C prevent and treat coronavirus disease 2019 (COVID-19)?. Med. Drug Discov..

[32] Schönrich G., Raftery M.J., Samstag Y. (2020). Devilishly radical NETwork in COVID-19: Oxidative stress, neutrophil extracellular traps (NETs), and T cell suppression. Adv. Biol. Regul..

[33] Jeon S., Ko M., Lee J., Choi I., Byun S.Y., Park S., Shum D., Kim S. (2020). Identification of Antiviral Drug Candidates against SARS-CoV-2 from FDA-Approved Drugs. Antimicrob. Agents Chemother..

[34] De Flora S., Balansky R., La Maestra S. (2020). Rationale for the use of N-acetylcysteine in both prevention and adjuvant therapy of COVID-19. FASEB J..

[35] Khomich O.A., Kochetkov S.N., Bartosch B., Ivanov A.V. (2018). Redox Biology of Respiratory Viral Infections. Viruses.

[36] Grunewald M.E., Fehr A.R., Athmer J., Perlman S. (2018). The coronavirus nucleocapsid protein is ADP-ribosylated. Virology.

[37] Xu Y.Z., Thuraisingam T., de Lima Morais D.A., Rola-Pleszczynski M., Radzioch D. (2010). Nuclear translocation of beta-actin is involved in transcriptional regulation during macrophage differentiation of HL-60 cells. Mol. Biol. Cell.

[38] Begemann H., Rastetter J., Begemann H., Rastetter J. (1972). Staining Methods. Atlas of Clinical Haematology.

[39] Zhang X., Goncalves R., Mosser D.M. (2008). The isolation and characterization of murine macrophages. Curr. Protoc. Immunol..

[40] Große M., Ruetalo N., Businger R., Rheber S., Setz C., Rauch P., Auth J., Brysch E., Schindler M., Schubert U. (2020). Evidence That Quinine Exhibits Antiviral Activity against SARS-CoV-2 Infection In Vitro. Preprints.

