# Supplementary material for: Immune-Modulating Drug MP1032 with SARS-CoV-2 Antiviral Activity In Vitro: A potential Multi-Target Approach for Prevention and Early Intervention Treatment of COVID-19
Source: Int J Mol Sci. 2020 Nov 20;21(22):8803. doi: 10.3390/ijms21228803 (PMC7699954; doi:10.3390/ijms21228803)
Supplement: Supplementary file 1 [file ijms-21-08803-s001.pdf]

Supplementary Materials

Immune-Modulating Drug MP1032 with SARS-CoV-2 Antiviral Activity In Vitro: A potential Multi-Target Approach for Prevention and Early Intervention Treatment of COVID-19

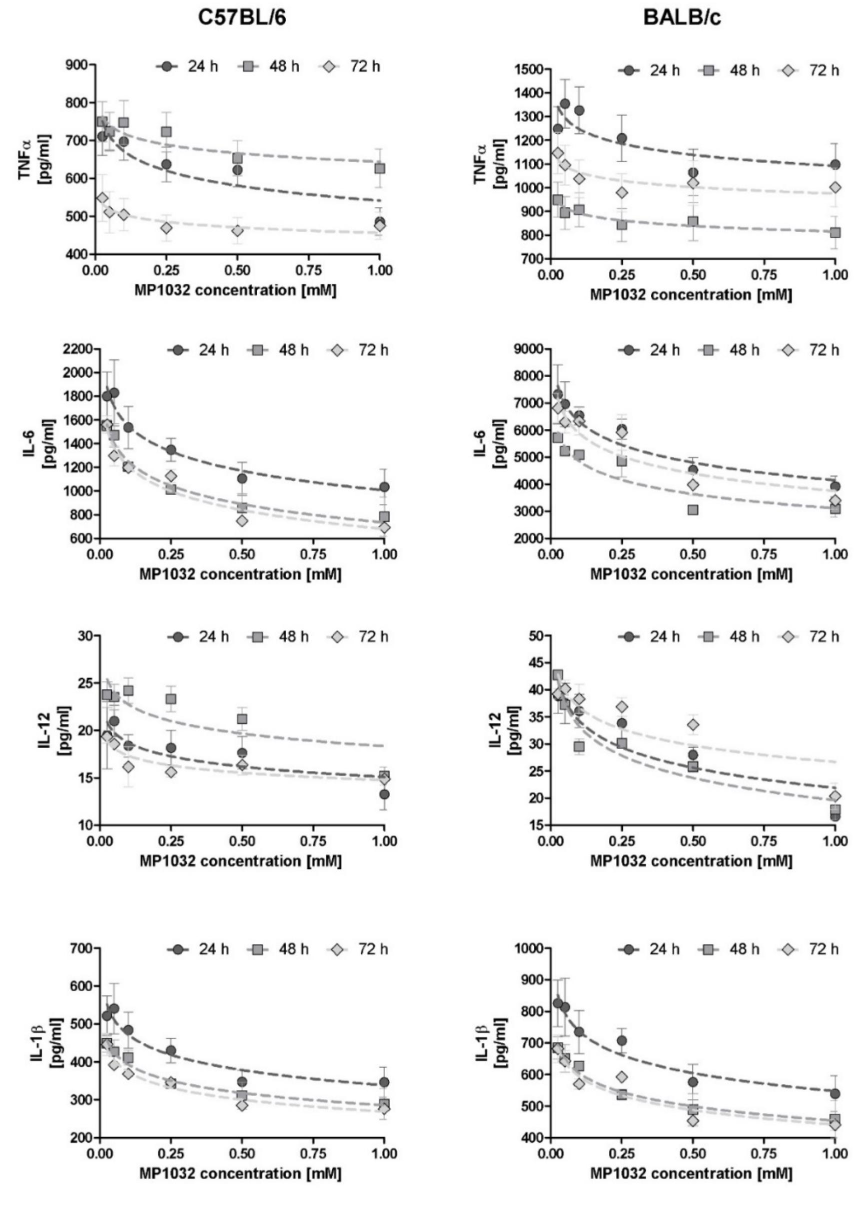

Figure S1. Dose-dependency of MP1032 treatment in murine peritoneal macrophages after 24, 48 or 72 h. Murine peritoneal macrophages were isolated from female C57Bl/6 and BALB/c mice four days after i.p. injection of 3% thioglycolate medium. One hour prior to LPS stimulation (0.1 µg/mL), isolated cells were pre-treated with different MP1032 concentrations (ranging from 1 mM to 0.025 mM). Cell-free supernatants were collected 24, 48, and 72 h after LPS stimulation and secreted cytokines were detected by ELISA. Data are expressed as mean ± SEM; n = 2.

Table S1. Tabulated List of Adverse Drug Reactions.Frequency classifications: very common (≥1/10), common (≥1/100 to <1/10), uncommon (≥1/1000 to <1/100), rare (≥1/10,000 to <1/1000), very rare (<1/10,000). Coded with MedDRA version 23.0.

System Organ Class (SOC)	Adverse Reaction (PT–Preferred Term)	Seriousness	Severity	Frequency
Blood and lymphatic system disorders	neutropenia	not serious	moderate	uncommon (1/146)
Cardiac disorders	palpitations	not serious	moderate	uncommon (1/146)
Gastrointestinal disorders	abdominal pain upper	not serious	mild	uncommon (1/146)
General disorders and administration site conditions	fatigue	not serious	mild	uncommon (1/146)
	feeling drunk	not serious	mild	uncommon (1/146)
	influenza-like illness	not serious	moderate	uncommon (1/146)
Infections and infestations	cystitis	not serious	moderate	uncommon (1/146)
	nasopharyngitis	not serious	moderate	common (2/146)
Nervous system disorders	headache	not serious	mild	uncommon (1/146)
Skin and subcutaneous tissue disorders	pruritus	not serious	mild to moderate	common (2/146)
	psoriasis	not serious	mild to severe	common (2/146)
